# Corneal Biomechanics and Other Factors Associated with Postoperative Astigmatism after Cataract Surgery

**DOI:** 10.3390/life14060655

**Published:** 2024-05-21

**Authors:** Kata Čulina, Martina Tomić, Tomislav Bulum, Aleksej Medić, Ivan Šoša, Kristina Ivanišević, Tomislav Jukić

**Affiliations:** 1 Eye Clinic “Medić, Jukić”, 21000 Split, Croatia; 2 Vuk Vrhovac University Clinic for Diabetes, Endocrinology and Metabolic Diseases, Merkur University Hospital, 10000 Zagreb, Croatia; 3 School of Medicine, University of Zagreb, 10000 Zagreb, Croatia; 4 School of Medicine, University of Rijeka, 51000 Rijeka, Croatia; 5 Ophthalmological Office “Ante Vuković”, 21300 Makarska, Croatia; 6 Department of Ophthalmology, Zagreb University Hospital Center, 10000 Zagreb, Croatia

**Keywords:** corneal hysteresis, corneal resistance factor, cataract surgery, preoperative and postoperative astigmatism

## Abstract

This study aimed to investigate the impact of the cornea’s biomechanical properties, corneal hysteresis (CH), and corneal resistance factor (CRF) on postoperative astigmatism after cataract surgery and determine the other factors that influence it. Forty eyes of 40 patients (13M/27F; the median age of 74) were included in this prospective study, underwent 2.75 mm incision cataract surgery, and were followed for 30 days. Visits were scheduled at baseline before surgery (V0), the 1st (V1), the 7th (V2), and the 30th (V3) postoperative days. The main parameters estimated and analyzed with Statistica^®^ 14.0.1 were CH, CRF, astigmatism diopter, and axis. Following the cataract surgery, the CH did not significantly change during the study visits (*p* = 0.109). However, there was a significant change in the CRF from baseline during the study visits (per protocol set) (*p* = 0.002). After a slight but insignificant increase from V0 to V1, post hoc analysis found a significant decrease in the mean CRF from V1 to V2 (*p* = 0.049) with no substantial change from V2 to V3. According to the post hoc analysis, the median astigmatism diopter increased significantly only from V0 to V1 (*p* = 0.001) and slightly but not significantly decreased to the end of the study with the achievement of a near-baseline value. The main predictors for the final astigmatism diopter (R^2^ = 0.898) obtained by stepwise regression analysis were its values at V0, V1, and V2 (*p* < 0.001). The CRF at V1 was marginally significant, with a negative parameter estimate of −0.098303 (*p* = 0.0623). In conclusion, there was no correlation between preoperative CH and CRF and postoperative astigmatism using 2.75 mm incision cataract surgery. However, the final astigmatism diopter’s main predictors were its baseline values before cataract surgery, the first, and the seventh postoperative days.

## 1. Introduction

Cataract surgery today is no longer just removing a clouded eye lens but also a refractive procedure aiming to achieve emmetropia. Choosing the appropriate intraocular lens (IOL) is one of the essential factors in achieving the best possible refractive results. Another equally important factor is the size of the surgical incision, since it can affect postoperative astigmatism, a significant cause of poor uncorrected visual acuity after cataract surgery. Today’s microincision cataract surgery minimizes the prevalence of postoperative surgically induced astigmatism (SIA) [1,2].

Except for the incision size, SIA also depends on other factors. The factors that have been mainly studied so far are the position and architecture of the incision. Regarding the position of the incision, there are corneal, limbal, scleral, and corneoscleral incisions. The most frequently used approach in modern cataract surgery is a corneal incision, and the postoperative astigmatism is smaller with increasing distance from the cornea [3,4]. Most studies suggest that the temporal position of the incision results in smaller surgically induced astigmatism if there is mild preoperative astigmatism and against-the-rule astigmatism [5,6,7]. In contrast, the superior position is more adequate for more significant preoperative astigmatism and with-the-rule astigmatism [8,9]. Regarding the architecture of the incision, there are several incision forms: uniplanar, biplanar, triplanar, and hinged corneal incision [10]. The incision architecture is essential for wound stability, which implies proper adhesion of incision edges, protection from infection, adequate wound healing, and reduction in surgically induced astigmatism [11,12].

However, the cornea’s biological influence is still insufficiently understood, but it may play a role in postoperative astigmatism. The cornea’s biological factors are its biomechanical properties: corneal hysteresis (CH) and corneal resistance factor (CRF) [13,14,15]. The CH refers to the cornea’s elastic properties, that is, its ability to return to its original position after temporary deformation, and the CRF indicates the cornea’s resistance to stress. The correlation between CH, CRF, and SIA is still unclear and dependent on other factors.

The biomechanical properties of the cornea may relate to SIA because cataract surgery causes a kind of stress in the cornea. In a clinical setting, it would be important to better understand the cornea’s reaction to cataract surgery and to detect whether and how much cataract surgery affects the cornea’s biomechanical properties and the cornea’s postoperative return to its original state. This study aimed to investigate the impact of the cornea’s biomechanical properties, CH and CRF, and other factors on postoperative astigmatism after cataract surgery.

## 2. Patients and Methods

### 2.1. Study Design and Patients

This prospective, observational cohort study was conducted at the Eye Clinic “Medic, Jukic” in Split, Croatia, following the Declaration of Helsinki, and was approved by the Eye Clinical Ethics Committee. The study’s patients received written and oral information about the study and signed written informed consent.

Forty patients (13 males and 27 females) referred to the Eye Clinic because of low visual acuity were included in this study, underwent cataract surgery, and were followed for 30 days. All included patients had best-corrected visual acuity (BCVA) ≤ 0.5 and immature cataracts graded as C2N3P2 to C4N4P4 according to the Lens Opacities Classification System (LOCS) [16]. Exclusion criteria were ocular surface disease, i.e., dry eye disease (Schirmer test < 10 mm, tear break-up time (TBUT) < 5 s, fluorescein positive test), pathology of the cornea, glaucoma, ocular hypertension, high refractive errors (myopia > −6 Dsph, hypermetropia > +5 Dsph), pseudoexfoliative syndrome, and intraoperative and postoperative complications.

### 2.2. Scheduled Visits and Ophthalmological Examinations

The first baseline visit (V0) was the inclusion visit in the study, performed before the cataract surgery, while the three further follow-up visits were scheduled on the first (V1), seventh (V2), and thirtieth postoperative days (V3).

At the first baseline visit, all patients underwent standard ophthalmological examinations and special diagnostics. Standard examinations included Snellen visual acuity and refraction testing, Goldman applanation tonometry, slit-lamp examination of the anterior eye segment in the physiological state and after mydriasis with eye drops containing 0.5% tropicamide, and indirect slit-lamp fundoscopy, both performed using the Zeiss Slit-lamp (Carl Zeiss Meditec AG, Jena, Germany).

After standard examinations, special diagnostics included corneal topography using the WaveLight Oculyzer™ II diagnostic system (Alcon, Freiburg im Breisgau, Germany) for the determination of corneal astigmatism diopter and axis, measurement of CH and CRF by the Ocular Response Analyzer G3 (ORA G3) (Reichert Inc., Buffalo, NY, USA), and examination at the Zeiss IOL Master 700 (Carl Zeiss Meditec AG, Jena, Germany), the first swept-source OCT-based biometer that enables OCT imaging and visualization of the entire length of the eyeball to determine the appropriate intraocular lens. Surgically induced astigmatism (SIA) diopter and axis were calculated using the corneal SIA tool available online on the American Society of Cataract and Refractive Surgery website (https://ascrs.org/tools/corneal-sia-tool (accessed on 1 March 2024)) [17].

At the further three follow-up visits (per protocol set), all patients underwent the following clinical and diagnostic examinations: Snellen visual acuity and refraction testing, corneal topography, corneal biomechanics (CH and CRF) measurements, and SIA diopter and axis calculation. All examinations and measurements at the first baseline and further follow-up visits were performed by the first author of this manuscript.

### 2.3. Measurement of Corneal Hysteresis and Corneal Resistance Factor

With its electro-optical system, the Ocular Response Analyzer (ORA) allows cornea-compensated intraocular pressure (IOP) measurements and can estimate CH and CRF [18,19]. It is designed to improve the accuracy of the IOP measurement by using corneal biomechanical data to calculate a biomechanically adjusted estimate of IOP. ORA measures the IOP at the moment when the deformation (i.e., applanation) of the cornea begins (P1) and the IOP at the moment when the deformation (i.e., applanation) ends (P2). During applanation, the cornea absorbs part of the energy; because of this, there is a difference in the initial and final applanation pressures, respectively. The pressure at which the applanation ends is lower than when the applanation begins. This pressure difference, P1–P2, represents the cornea’s elastic property or CH and is measured in mmHg. A higher CH indicates a greater ability of the cornea to return to its initial state after applying stress. The CRF represents the cornea’s resistance to stress and is calculated using the formula CRF = P1 − kP2, where k denotes a constant obtained by empirical evaluation of the relationship between the initial applanation intraocular pressure (P1), final applanation intraocular pressure (P2), and central corneal thickness. The CRF is also measured in mmHg. A higher CRF indicates a higher cornea’s resistance to permanent deformation after applied stress.

### 2.4. Cataract Surgery

Cataract surgery was performed by a single operator, the last author of this manuscript, using the ophthalmic microscope OPMI Lumera 700 (Carl Zeiss Meditec AG, Jena, Germany) and phacoemulsification device Centurion Vision System (Alcon Laboratories, Inc., Fort Worth, TX, USA). The operation was performed through a 2.75 mm corneal incision positioned at 12 o’clock in the superior position using the phacoemulsification method with IOL implantation in the capsular bag. Postoperatively, all patients received the same therapy: Maxitrol Eye Drops four times a day and Maxitrol Eye Ointment once a day for the first seven days, and then Maxitrol Eye Drops three times a day for the next two weeks.

### 2.5. Statistical Analysis

Statistical analysis was performed, and the graphs were created using the statistical package Statistica™ 14.0.1. In all analyses, a *p*-value < 0.05 was considered statistically significant. After testing the normality of the data distribution with the Shapiro–Wilk test, normally distributed continuous variables were expressed as mean ± SD and non-normally distributed variables as median (min, max). The categorical variable (gender) was presented as a number. Differences in the distributions of dependent continuous data were evaluated by parametric (repeated measures ANOVA) and nonparametric (Friedman ANOVA) tests. A nonparametric test was used when the assumption of homogeneity of variance for the tested variables was not met. The Scheffe and Wilcoxon tests were used for post hoc analyses. The Spearman rank correlation test was used to evaluate the relationship between the studied variables, and stepwise regression was used to detect the main predictors of the final astigmatism diopter.

## 3. Results

Forty eyes of 40 patients, with a median age of 74 (min 48, max 83) years, were included in this study. Their baseline characteristics at the inclusion visit before cataract surgery (V0) are presented in Table 1.

**Figure 1:**
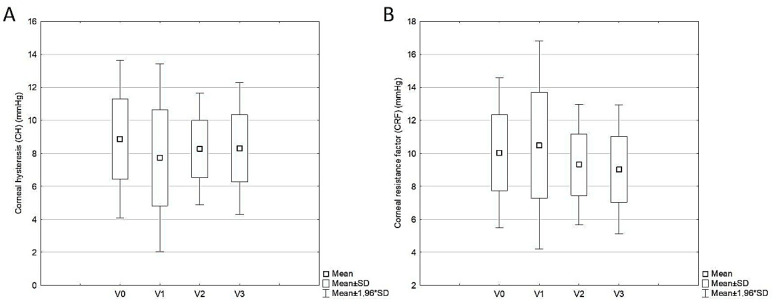
Change in corneal hysteresis (**A**) and corneal resistance factor (**B**) from baseline during the study visits in 40 study eyes.

Following the cataract surgery, the CH did not significantly change during the study visits (*p* = 0.109) (Table 2, Figure 1A). However, there was a significant change in the CRF from baseline during the study visits (per protocol set) in 40 study eyes (*p* = 0.002) (Table 2, Figure 1B). According to the post hoc analysis, the mean CRF decreased significantly from the first (V1) to the second postoperative visit (V2) (10.49 ± 3.22 vs. 9.31 ± 1.87, *p* = 0.049), after a slight but insignificant increase from baseline (V0) to the first postoperative visit (V1) (*p* = 0.761). However, no substantial change in the mean CRF was observed from the second (V2) to the third postoperative visit (V3) (*p* = 0.933).

BCVA increased significantly from baseline during the study visits (per protocol set) (*p* < 0.001) (Table 2). The post hoc analysis also showed a significant increase in BCVA between each study visit (V0–V1 *p* < 0.001, V1–V2 *p* < 0.001, V2–V3 *p* = 0.019). The spherical equivalent (SE) refraction decreased significantly during the study period (*p* = 0.033). However, the post hoc analysis found a marginal decrease in the SE between V0 and V1 (*p* = 0.058) and a significant decline between V2 and V3 (*p* < 0.001).

**Figure 2:**
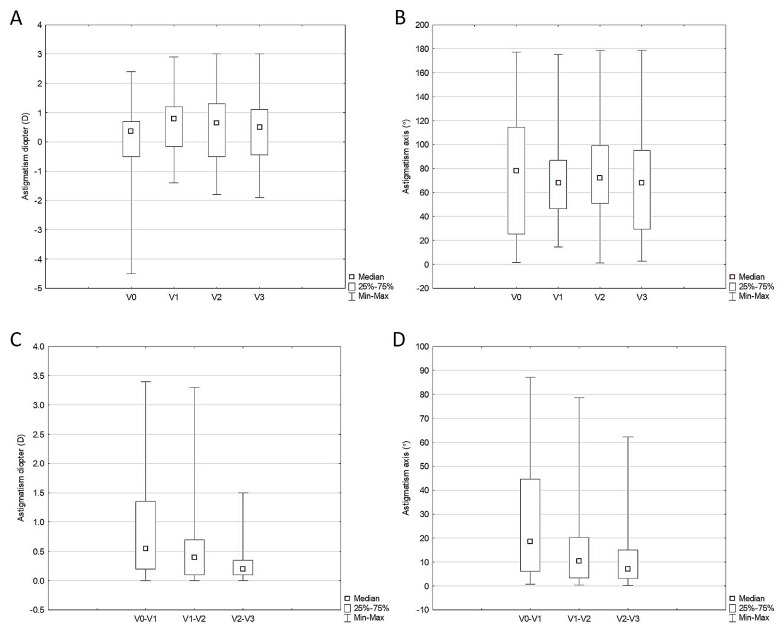
Change in astigmatism diopter (**A**) and axis (**B**) from baseline during the study visits in 40 study eyes and the amplitudes of their changes (**C**,**D**) between study visits.

As with the CRF, the diopter of corneal astigmatism determined by corneal topography significantly changed in 40 eyes from baseline to the study visits (as planned) (*p* = 0.009) (Table 2, Figure 2A). However, according to the post hoc analysis, the median diopter of astigmatism changed significantly only from the baseline (V0) to the first postoperative visit (V1) (0.4 D vs. 0.8 D, *p* = 0.001). In contrast, there were no significant changes in the median diopter of astigmatism from the first (V0) to the second (V2) (*p* = 0.139) or from the second (V2) to the third postoperative visit (V3) (*p* = 0.863). The median degree of the corneal astigmatism axis determined by corneal topography did not significantly change from the study’s baseline to each visit in 40 study eyes (Table 2, Figure 2B). However, the amplitudes of corneal astigmatism diopter and axis changes were the highest between baseline (V0) and the first postoperative visit (V1), decreasing gradually until the study’s end (Figure 2C,D). In contrast to the diopter of corneal astigmatism determined by corneal topography, the SIA diopter did not significantly differ throughout the study (*p* = 0.741) (Table 2). Also, no differences in the SIA axis were observed during the study period (*p* = 0.312) (Table 2).

Significantly positive, moderate to very good, or excellent correlations were seen between the CH and CRF at the beginning of the study, before cataract surgery, and at all other study visits (Table 3).

Nevertheless, no significant relation was found between corneal characteristics (CH and CRF) and the diopter and axis of corneal astigmatism at baseline and all other study visits (*p* > 0.05) (data not shown in table). Also, no relationships were observed between the baseline preoperative and all postoperative study visit values of CH, CRF, SIA diopter, and SIA axis ((*p* > 0.05) (data not shown in table).

The diopter of corneal astigmatism at the final visit was significantly positive or very good to excellent and associated with its values at baseline before cataract surgery and other study visits (*p* < 0.001) (Table 4). However, the amplitude of corneal astigmatism change from baseline (V0) to the final visit (V3) related significantly positively only to the amplitude of the astigmatism change between baseline (V0) and the first postoperative visit (V1) (*p* < 0.001), while the other relations were not significant (*p* > 0.05) (Table 5).

The main predictors for the final diopter of corneal astigmatism (R^2^ = 0.898) obtained by stepwise regression analysis were its values at baseline before cataract surgery (V0), the first (V1), and the seventh postoperative day (V2) (Table 6). Besides all those listed, the CRF at the first postoperative day (V1) was found to be marginally significant, with the negative parameter estimate of −0.098303 (*p* = 0.0623) influencing the final astigmatism diopter value relative to its one-unit change and representing the marginal impact of the weaker corneal characteristic (weaker resistance of the cornea to stress) on postoperative astigmatism.

## 4. Discussion

Cataract surgery with sophisticated intraocular lens implantation requires the most accurate prediction of postoperative refractive results. The present study tried to establish whether the biomechanical properties of the cornea influence these results and to determine which other factors have a role in postoperative astigmatism after cataract surgery.

Only a few studies have examined the association between the cornea’s biomechanical properties and SIA. The first published study was by Denoyer et al. [20]. They found a positive correlation between incision size and SIA and a negative correlation between preoperative CH, CRF, and SIA values. The present study did not find a correlation between the cornea’s biomechanical properties (CH and CRF) and the diopter and axis of corneal astigmatism and SIA. However, this study’s results indicate that CH and CRF values changed after cataract surgery. The CH decreased marginally, and the CRF increased marginally from baseline to the first postoperative day, while both variables gradually recovered in the following days. The CH recovered better than the CRF on the 30th postoperative day, but both returned to near-preoperative values.

Denoyer et al., in their study, monitored changes in CH and CRF after cataract surgery in two groups of patients [20]. In one group, cataract surgery was performed through a small incision (2.75 mm), while in the other group, it was performed through a microincision (≤2.2 mm). In their study, CH and CRF decreased postoperatively. As in the present study, CH recovers entirely on the 30th postoperative day. However, regarding CRF, there are some discrepancies between the present study and the study of Denoyer et al. Denoyer et al. found a recovery of CRF to preoperative values in the microincision group. In the small incision group, when a step-constructed triplanar incision was performed, the CRF recovered rapidly, while when other uniplanar and biplanar incisions were completed, the CRF was significantly reduced. Based on these results, the authors suggested that the stepped triplanar construction of the incision is a better choice in cataract surgery because it does not weaken the corneal resistance [20].

Zheng et al., in their study, examined the changes in CH and CRF after cataract surgery performed through 2.2 mm and 3.0 mm incisions. The incisions were made biplanarly at the 10 o’clock position with a distance of 0.5 mm from the limbus. While CH values fluctuated, as in the study of Denoyer et al., CRF values did not change significantly during the four weeks after surgery [21].

Pniakowska et al. analyzed the changes in CH and CRF after cataract surgery through a 2 mm incision at the 12 o’clock position without specifying the architectural type of the incision in two groups of patients: group 1 with corneal astigmatism values of less than +1.0 dcyl and group 2 with values between +1.0 dcyl and +2.25 dcyl. Their results showed a postoperative decrease in CH and CRF values in the first group of patients. The CH recovered to preoperative values, and the CRF remained significantly reduced one month after surgery. In the second group, the CH and CRF remained significantly lowered one month after cataract surgery [22].

In the present study, cataract surgery was performed through a 2.75 mm biplanar incision, and as previously mentioned, we noted a slight but statistically insignificant increase in CRF values in the 1st postoperative day, with a significant decrease till the 7th, and a gradual insignificant reduction toward the 30th postoperative day. This decrease in CRF values on the 30th postoperative day contradicts the results of Zheng et al. but coincides with the results of the studies by Denoyer et al. and Pniakowska et al. Following Denoyer et al.’s thinking about the influence of the architecture of the incision on corneal resistance, it might be stated that the small biplanar incision leads to a certain degree of weakening of the corneal resistance.

Usually, SIA positively correlates with incision size, and larger incisions induce more astigmatism. An incision width of 1.8–2.2 mm has a relatively small SIA [23]. Astigmatic effects have been reduced after decreasing the incision size from 3.2 mm to 2.2 mm or 1.8 mm. A study that included 146 eyes found that a corneal incision of 2.75 mm for cataract surgery, using either a temporal or superior approach, induces little astigmatism change in eyes [24]. Another study that included 100 eyes of 50 patients who underwent bilateral intraocular lens implantation through a 2.2 mm or 2.75 mm corneal incision found that a lower incision size of 2.2 mm compared to 2.75 mm did not result in less SIA [25]. In addition, a study that investigated the changes in incision sizes after implantation of an intraocular lens with a mean incision size of 2.27 mm found that the CH and CRF values were not correlated with the final incision size, and the CH was more predictive of postoperative surgically induced astigmatism than incision size [26].

The present study did not find a correlation between corneal biomechanical properties and postoperative astigmatism after cataract surgery. However, as in the CRF value, there was a significant change in the diopter of corneal astigmatism from baseline during the study visits (per protocol set). These two variables changed similarly; the mean CRF and the median diopter of corneal astigmatism increased from baseline to the first postoperative day, while after that, they both decreased in the 7th and 30th postoperative days.

The increase in median diopter of corneal astigmatism after cataract surgery is because of a corneal shape change due to wounds at the incision site and postoperative corneal edema [27]. Regarding CRF, while in most studies, it decreases postoperatively or remains the same, in this study, we note a slight increase on the first postoperative day. Similar behavior of the cornea was described in the study of Alió et al. [28]. They registered a postoperative increase in CRF, followed by its decrease, after cataract surgery in the group with microincision, while in the group with coaxial phacoemulsification, they did not find a significant change.

Postoperative astigmatism can also be influenced by corneal thickness [29]. The cornea is thicker in the vertical than the horizontal direction, so the posterior cornea surface is more astigmatic than the anterior corneal surface. The cornea is also thinner in older age [30]. However, pericentral corneal thickness is significantly greater in the superior quadrant and not affected by aging compared to the inferior, nasal, and temporal quadrants [29]. In this study, we did not measure the corneal thickness, and the surgery was performed through a 2.75 mm corneal incision at 12 o’clock in the superior position. This area has the thickest cornea compared to other places, and corneal thickness is negatively related to corneal astigmatism [31].

Different corneal incision positions have different risks of SIA because of the different distances of each position to the central visual axis [32]. Common incision positions include temporal, superior, and nasal. In the present study, we used a corneal incision positioned at 12 o’clock in the superior position. Temporal and nasal incisions postoperatively have similar corneal and astigmatic changes and induce a lower degree of SIA compared to the superior position. Superior positions have longer proximity of the superior limbus to the central visual axis and are recommended for with-the-rule and negligible astigmatism [33].

Several limitations of the present study must be mentioned. Similar changes in median diopter of corneal astigmatism and CRF observed in this study could be explained by postoperative changes in corneal thickness. A corneal pachymeter measures corneal thickness, a sensitive indicator of endothelial physiology that correlates well with functional measurements. However, this study did not monitor corneal thickness, which is a limiting factor. Second, the operation was performed only through a 2.75 mm corneal incision, so we could not compare the impact on CH and CRF values with different incision sizes. Third, different corneal incision positions (superior used in this study, temporal) have different risks of postoperative astigmatism. Finally, the sample size in this study was small, reducing the study’s power.

## 5. Conclusions

Postoperative astigmatism is a significant cause of poor visual acuity after cataract surgery. In this short-term follow-up of one month postoperatively, there was no correlation between preoperative CH and CRF and postoperative astigmatism using 2.75 mm superior incision cataract surgery. However, the main predictors for the final diopter of astigmatism were its values at baseline before cataract surgery, the first, and the seventh postoperative day. The discrepancy with the results of several studies conducted so far points to the need for additional research on a larger number of patients in order to better understand the cornea’s reaction to cataract surgery and thus achieve more precise postoperative results.

## Figures and Tables

**Table 1:** Baseline characteristics of 40 patients (eyes) included in the study.

Characteristics	Patients/Eyes (n = 40)
CH (mmHg) *	8.86 ± 2.44
CRF (mmHg) *	10.04 ± 2.32
BCVA (decimal) *	0.29 ± 0.18
Refraction (SE) **	−0.75 (−6.0, 3.5)
Astigmatism (D) **	0.4 (−4.5, 2.4)
Axis (°) **	78.2 (1.6, 177.3)

* mean ± SD. ** median (min, max). Abbreviations: CH—corneal hysteresis, CRF—corneal resistance factor, BCVA—best-corrected visual acuity, SE—spherical equivalent, D—diopter of corneal astigmatism determined by corneal topography, °—degrees of corneal astigmatism axis determined by corneal topography.

**Table 2:** Changes in the analyzed characteristics from baseline during the study visits (per protocol set) in 40 study patients (eyes).

Characteristics	V0	V1	V2	V3	*p*
CH (mmHg) *	8.86 ± 2.44	7.73 ± 2.91	8.26 ± 1.73	8.30 ± 2.04	0.109 ^a^
CRF (mmHg) *	10.04 ± 2.32	10.49 ± 3.22	9.31 ± 1.87	9.03 ± 1.99	0.002 ^a^
BCVA (decimal) *	0.29 ± 0.18	0.49 ± 0.19	0.83 ± 0.12	0.92 ± 0.09	<0.001 ^a^
Refraction (SE) **	−0.75 (−6.0, 3.5)	−0.37 (−1.5, 1.0)	−0.5 (−1.0, 0.5)	−0.2 (−0.75, 0.75)	0.033 ^b^
Astigmatism (D) **	0.4 (−4.5, 2.4)	0.8 (−1.4, 2.9)	0.7 (−1.8, 3.0)	0.5 (−1.9, 3.0)	0.009 ^b^
Axis (°) **	78.2 (1.6, 177.3)	67.9 (1.4, 175.2)	72.2 (1.3, 178.7)	68.0 (2.6, 178.8)	0.861 ^b^
SIA (D) **	-	0.79 (0.11, 4.24)	0.69 (0.1, 3.76)	0.63 (0.1, 4.06)	0.741 ^b^
SIA (°) **	-	132.0 (3, 177)	125.5 (3, 174)	113.5 (1.7, 177)	0.312 ^b^

* mean ± SD. ** median (min, max). ^a^ Repeated measures ANOVA test. ^b^ Friedman ANOVA test Abbreviations: CH—corneal hysteresis, CRF—corneal resistance factor, BCVA—best-corrected visual acuity, SE—spherical equivalent, Astigmatism (D)—diopter of corneal astigmatism determined by corneal topography, Axis (°)—degrees of astigmatism axis, SIA (D)—diopter of surgically induced astigmatism, SIA (°)—degree of surgically induced astigmatism axis.

**Table 3:** Correlations between corneal hysteresis and corneal resistance factor from baseline during the study visits (per protocol set) in 40 study eyes.

		Corneal Hysteresis				Corneal Resistance Factor			
		V0	V1	V2	V3	V0	V1	V2	V3
Corneal hysteresis	V0	1	0.046	0.348 *	0.498 *	0.889 **	0.217	0.441 *	0.595 **
	V1		1	0.247	0.177	0.103	0.499 *	0.345 *	0.239
	V2			1	0.560 **	0.451 *	0.223	0.809 **	0.643 **
	V3				1	0.445 *	0.175	0.506 **	0.864 **
Corneal resistance factor	V0					1	0.349 *	0.620 **	0.631 **
	V1						1	0.399 *	0.318 *
	V2							1	0.708 **
	V3								1

* *p* < 0.05. ** *p* < 0.001. Spearman rank correlation test.

**Table 4:** Correlations between the diopter of corneal astigmatism at final (V3), baseline (V0), and other study visits (V1, V2) in 40 study eyes.

		Diopter of Corneal Astigmatism at V3		
		Spearman R	t (N-2)	*p*
Diopter of corneal astigmatism	V0	0.736	6.7	<0.001
	V1	0.639	5.133	<0.001
	V2	0.894	12.267	<0.001

**Table 5:** Correlations between the amplitude of corneal astigmatism change from V0 to V3 and the amplitude of astigmatism changes at intermediate times during the study.

		Amplitude of Corneal Astigmatism Change between V0 and V3		
		Spearman R	t (N-2)	*p*
Amplitude of corneal astigmatism changes	V0–V1	0.525	3.803	<0.001
	V1–V2	0.203	1.276	0.209
	V2–V3	−0.062	−0.381	0.705

**Table 6:** Results of stepwise regression analysis for the final diopter of corneal astigmatism as a dependent variable.

Variable	Estimate	Standard Error	F	*p*	Adjusted R^2^	R^2^
CRF—V1	−0.098	1.028	3.69	0.0623	0.0645	0.898
Astigmatism—V0	0.504	0.759	38.55	0.0000	0.4905	
Astigmatism—V1	0.497	0.764	37.49	0.0000	0.4834	
Astigmatism—V2	0.898	0.345	333.07	0.0000	0.8949	

Abbreviations: CRF—corneal resistance factor.

## Data Availability

The data presented in this study are available on a specific request from the corresponding author.

## References

[1] Liang J.L., Xing X.L., Yang X.T., Jiang Y.F., Zhang H. (2019). Clinical comparison analysis in surgically induced astigmatism of the total, anterior and posterior cornea after 2.2-mm versus 3.0-mm clear corneal incision cataract surgery. Zhonghua Yan Ke Za Zhi.

[2] Pattanayak S., Mathur S., Nanda A.K., Subudhi B.N.R. (2022). Postoperative astigmatic considerations in manual small-incision cataract surgery—A review. Indian J. Ophthalmol..

[3] Sonmez S., Karaca C. (2020). The effect of tunnel length and position on postoperative corneal astigmatism: An optical coherence tomographic study. Eur. J. Ophthalmol..

[4] Hashemi H., Khabazkhoob M., Soroush S., Shariati R., Miraftab M., Yekta A. (2016). The location of incision in cataract surgery and its impact on induced astigmatism. Curr. Opin. Ophthalmol..

[5] Barequet I.S., Yu E., Vitale S., Cassard S., Azar D.T., Stark W.J. (2004). Astigmatism outcomes of horizontal temporal versus nasal clear corneal incision cataract surgery. J. Cataract Refract. Surg..

[6] Borasio E., Mehta J.S., Maurino V. (2006). Surgically induced astigmatism after phacoemulsification in eyes with mild to moderate corneal astigmatism: Temporal versus on-axis clear corneal incisions. J. Cataract Refract. Surg..

[7] Altan-Yaycioglu R., Akova Y.A., Akca S., Gur S., Oktem C. (2007). Effect on astigmatism of the location of clear corneal incision in phacoemulsification of cataract. J. Refract. Surg..

[8] Roman S., Givort G., Ullern M. (1997). Choice of the site of incision for cataract surgery without suture according to preoperative astigmatism. J. Fr. Ophtalmol..

[9] Archana S., Khurana A.K., Chawla U. (2011). A comparative study of sclero-corneal and clear corneal tunnel incision in manual small-incision cataract surgery. Nepal. J. Ophthalmol..

[10] Taban M., Rao B., Reznik J., Zhang J., Chen Z., McDonnell P.J. (2004). Dynamic morphology of sutureless cataract wounds—Effect of incision angle and location. Surv. Ophthalmol..

[11] Simşek S., Yaşar T., Demirok A., Cinal A., Yilmaz O.F. (1998). Effect of superior and temporal clear corneal incisions on astigmatism after sutureless phacoemulsification. J. Cataract Refract. Surg..

[12] Ernest P., Hill W., Potvin R. (2011). Minimizing surgically induced astigmatism at the time of cataract surgery using a square posterior limbal incision. J. Ophthalmol..

[13] Glass D.H., Roberts C.J., Litsky A.S., Weber P.A. (2008). A viscoelastic biomechanical model of the cornea describing the effect of viscosity and elasticity on hysteresis. Investig. Ophthalmol. Vis. Sci..

[14] Kotecha A. (2007). What biomechanical properties of the cornea are relevant for the clinician?. Surv. Ophthalmol..

[15] Chong J., Dupps W.J. (2021). Corneal biomechanics: Measurement and structural correlations. Exp. Eye. Res..

[16] Chylack L.T., Wolfe J.K., Singer D.M., Leske M.C., Bullimore M.A., Bailey I.L., Friend J., McCarthy D., Wu S.Y. (1993). The Lens Opacities Classification System III. Arch. Ophthalmol..

[17] Koch D.D., Wang L. (2015). Surgically induced astigmatism. J. Refract. Surg..

[18] Luce D.A. (2005). Determining in vivo biomechanical properties of the cornea with an ocular response analyzer. J. Cataract. Refract. Surg..

[19] Kaushik S., Pandav S.S. (2012). Ocular response analyzer. J. Curr. Glaucoma Pract..

[20] Denoyer A., Ricaud X., Van Went C., Labbé A., Baudouin C. (2013). Influence of corneal biomechanical properties on surgically induced astigmatism in cataract surgery. J. Cataract Refract. Surg..

[21] Zhang Z., Yu H., Dong H., Wang L., Jia Y.D., Zhang S.H. (2016). Corneal biomechanical properties changes after coaxial 2.2-mm microincision and standard 3.0-mm phacoemulsification. Int. J. Ophthalmol..

[22] Pniakowska Z., Jurowski P. (2019). Influence of preoperative astigmatism on corneal biomechanics and accurate intraocular pressure measurement after micro-incision phacoemulsification. Int. J. Ophthalmol..

[23] Yang J., Wang X., Zhang H., Pang Y., Wei R.H. (2017). Clinical evaluation of surgery-induced astigmatism in cataract surgery using 2.2 mm or 1.8 mm clear corneal micro-incisions. Int. J. Ophthalmol..

[24] Giansanti F., Rapizzi E., Virgili G., Mencucci R., Bini A., Vannozzi L., Menchini U. (2006). Clear corneal incision of 2.75 mm for cataract surgery induces little change of astigmatism in eyes with low preoperative corneal cylinder. Eur. J. Ophthalmol..

[25] Karmiris E., Chalkiadaki E., Papakonstantinou E., Georgalas I. (2022). Long-term clinical outcomes obtained with bilateral implantation of a multifocal intraocular lens through two different-sized corneal incisions. Saudi J. Ophthalmol..

[26] Guarnieri A., Moreno-Montañés J., Sabater A., Gosende-Chico I., Bonet-Farriol E. (2013). Final incision size after cataract surgery with toric intraocular lens implantation using 2 techniques. J. Cataract Refract. Surg..

[27] Alió J.L., Agdeppa M.C., Rodríguez-Prats J.L., Amparo F., Piñero D.P. (2010). Factors influencing corneal biomechanical changes after microincision cataract surgery and standard coaxial phacoemulsification. J. Cataract. Refract. Surg..

[28] Sheoran K., Arya S.K., Bansal R.K., Jinagal J., Jha U.P. (2022). Surgically induced astigmatism and posterior corneal curvature changes following phacoemulsification. Indian J. Ophthalmol..

[29] Ueno Y., Hiraoka T., Miyazaki M., Ito M., Oshika T. (2015). Corneal thickness profile and posterior corneal astigmatism in normal corneas. Ophthalmology.

[30] Ueno Y., Hiraoka T., Beheregaray S., Miyazaki M., Ito M., Oshika T. (2014). Age-related changes in anterior, posterior, and total corneal astigmatism. J. Refract. Surg..

[31] Zhu L., Yuan Z., Liang S., Zhao D., Zhou C. (2019). Relationship between corneal astigmatism and the distribution of corneal thickness along different principal meridians. Res. Square.

[32] Nikose A.S., Saha D., Laddha P.M., Patil M. (2018). Surgically induced astigmatism after phacoemulsification by temporal clear corneal and superior clear corneal approach: A comparison. Clin. Ophthalmol..

[33] Hayashi K., Sato T., Yoshida M., Yoshimura K. (2019). Corneal shape changes of the total and posterior cornea after temporal versus nasal clear corneal incision cataract surgery. Br. J. Ophthalmol..

